# Supplementary material for: Targeting carA Using Optimized Antisense Peptide Nucleic Acid–Cell-Penetrating Peptide Conjugates in Acinetobacter baumannii: A Novel Antibacterial Approach
Source: Int J Mol Sci. 2025 Sep 29;26(19):9526. doi: 10.3390/ijms26199526 (PMC12525168; doi:10.3390/ijms26199526)
Supplement: Supplementary file 1 [file ijms-26-09526-s001.zip › ijms-3884511-supplementary.pdf]

Table S1. Potential Gene Binding Sites in the *A. baumannii* ATCC17978 Genome (CP033110) for PNA Length of 10 nucleotides

No.	Gene	Function	Locus tag
1	-	Non-coding DNA sequences	-
2	-	aldo/keto reductase	EAV54_01120
3	*yccS*	TIGR01666 family membrane protein	EAV54_01780
4	-	acyl-CoA dehydrogenase	EAV54_01950
5	-	response regulator	EAV54_03475
6	-	poly alpha-glucosyltransferase	EAV54_04090
7	*carA*	carbamoyl-phosphate synthase small subunit	EAV54_04140
8	*xerC*	tyrosine recombinase	EAV54_04480
9	-	alpha/beta fold hydrolase	EAV54_06080
10	*rpoH*	RNA polymerase sigma factor	EAV54_06330
11	-	type 1 glutamine amidotransferase domain-containing protein	EAV54_06895
12	*murB*	UDP-N-acetylmuramate dehydrogenase	EAV54_07435
13	-	hypothetical protein	EAV54_08390
14	*clpA*	ATP-dependent Clp protease ATP-binding subunit	EAV54_08410
15	*adeT1*	putative multidrug efflux protein	EAV54_09245
16	-	aspartate ammonia-lyase	EAV54_09640
17	*lpdA*	Dihydrolipoyl dehydrogenase	EAV54_09760
18	-	ABC transporter permease	EAV54_09935
19	*gspI*	Type II secretion system protein	EAV54_10660
20	*katE*	catalase HPII	EAV54_11605
21	-	putative 2-aminoethylphosphonate ABC transporterpermease subunit	EAV54_11735
22	-	Non-coding DNA sequences	-
23	-	EamA family transporter	EAV54_11940
24	-	HlyD family type I secretion periplasmic adaptorsubunit	EAV54_12370
25	*terL*	helicase	EAV54_12950
26	-	amino acid permease	EAV54_13380
27	*rsmD*	16S rRNA (guanine(966)-N(2))-methyltransferase	EAV54_13730
28	-	DHA2 family efflux MFS transporter permease subunit	EAV54_14315
29	-	LysR family transcriptional regulator	EAV54_14610
30	*bamD*	Outer membrane protein assembly factor	EAV54_14660
31	*bioD*	ATP-dependent dethiobiotin synthetase	EAV54_14810

32	*ubiE*	Bifunctional demethylmenaquinone methyltransferase/2-methoxy-6-polyprenyl-1,4,-benzoquinol methylase	EAV54_16875
33	*murD*	UDP-N-acetylmuramoyl-L-alanine--D-glutamate ligase	EAV54_17400
34	*amt*	Ammonium transporter	EAV54_17520
35	*parC*	DNA topoisomerase IV subunit	EAV54_17650
36	-	beta-ketoacyl-ACP synthase I	EAV54_18220
37	*ribF*	bifunctional riboflavin kinase/FAD synthetase	EAV54_18535

Table S2. Potential Gene Binding Sites in the *A. baumannii* ATCC17978 Genome (CP033110) for control 1 (mismatch) PNA.

No.	Gene	Function	Locus tag
1	-	TolC family protein	EAV54_01415
2	*gltB*	Glutamate synthase large subunit	EAV54_01575
3	-	TonB-dependent siderophore receptor	EAV54_08350
4	-	TetR/AcrR family transcriptional regulator	EAV54_12185
5	-	Ferredoxin reductase	EAV54_18445

Table S3. Potential Gene Binding Sites in the *A. baumannii* ATCC17978 Genome (CP033110) for control 2 (mismatch) PNA.

No.	Gene	Function	Locus tag
1	*ftsH*	ATP-dependent zinc metallopeptidase	EAV54_04165
2	-	N-acetyltransferase	EAV54_05010
3	*trpD*	Anthranilate phosphoribosyltransferase	EAV54_05830
4	*umuC*	DNA polymerase V subunit	EAV54_07805
5	-	Aromatic ring-hydroxylating dioxygenase subunit alpha	EAV54_08645
6	-	DUF2184 domain-containing protein	EAV54_12910
7	-	Undecaprenyl-diphosphatase	EAV54_18680

Supplementary figures

PNA

Sample No(Name): 56 (2a-PNA(24)-021 / C240081)

Lot No : 241431

N-term C-term

Sequence : KFFKFFKFFK-tca aa^c ca^a agc t

(x^: Alpha-D-Arg)

Amount

OD(260nm): 2.8 **nmole:** 20.0 **Molecular Weight:** 5091.4 **Weight:** 101.8 µg

(Weight was calculated from OD value & Extinction coefficient at 260nm, ε_{260} $^{+}$= 137.9 mL/(µmol x cm))

+ ε_{260}(a) = 13.7mL/(µmolexcm), ε_{260}(g) = 11.7mL/(µmolexcm), ε_{260}(c) = 6.6mL/(µmolexcm), ε_{260}(t) = 8.8mL/(µmolexcm),

Analysis

HPLC

Instrument : Agilent 1100 Series (Agilent Technologies)

Purity : 99.9%

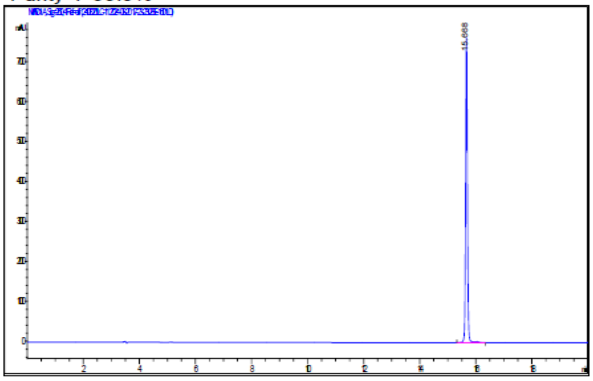

MALDI MS

Instrument: AXIMA-Assurance (Shimadzu Biotech)

MS Found (M+1) : 5093.5

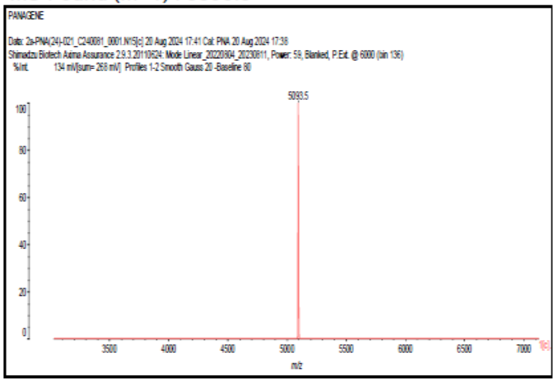

PNA

Sample No(Name): 09 (PNA59 / C240252)

Lot No : 241924

N-term C-term

Sequence : KFFKFFKFFK-aac^ caa a^gc t

(x^: Alpha-D-Arg)

Amount

OD(260nm): 2.2 **nmole:** 20.0 **Molecular Weight:** 4298.6 **Weight:** 85.9 µg

(Weight was calculated from OD value & Extinction coefficient at 260nm, ε_{260} $^{+}$= 108.8 mL/(µmol x cm))

+ ε_{260}(a) = 13.7mL/(µmolexcm), ε_{260}(g) = 11.7mL/(µmolexcm), ε_{260}(c) = 6.6mL/(µmolexcm), ε_{260}(t) = 8.8mL/(µmolexcm),

Analysis

HPLC

Instrument : Agilent 1100 Series (Agilent Technologies)

Purity : 99.9%

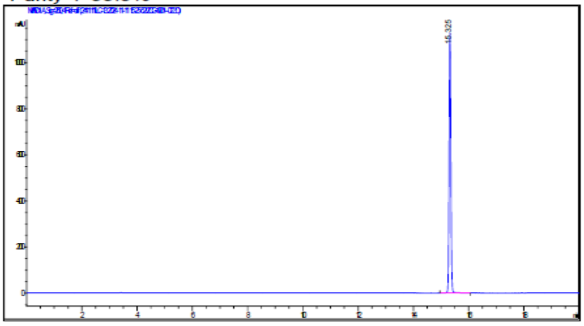

MALDI MS

Instrument: AXIMA-Assurance (Shimadzu Biotech)

MS Found (M+1) : 4299.0

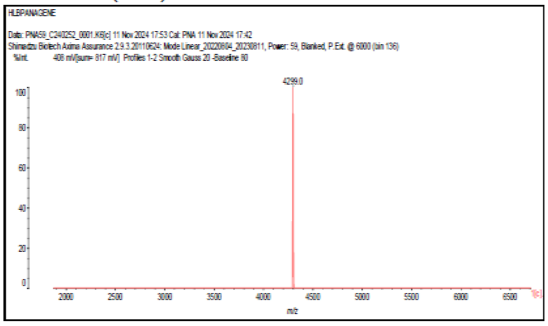

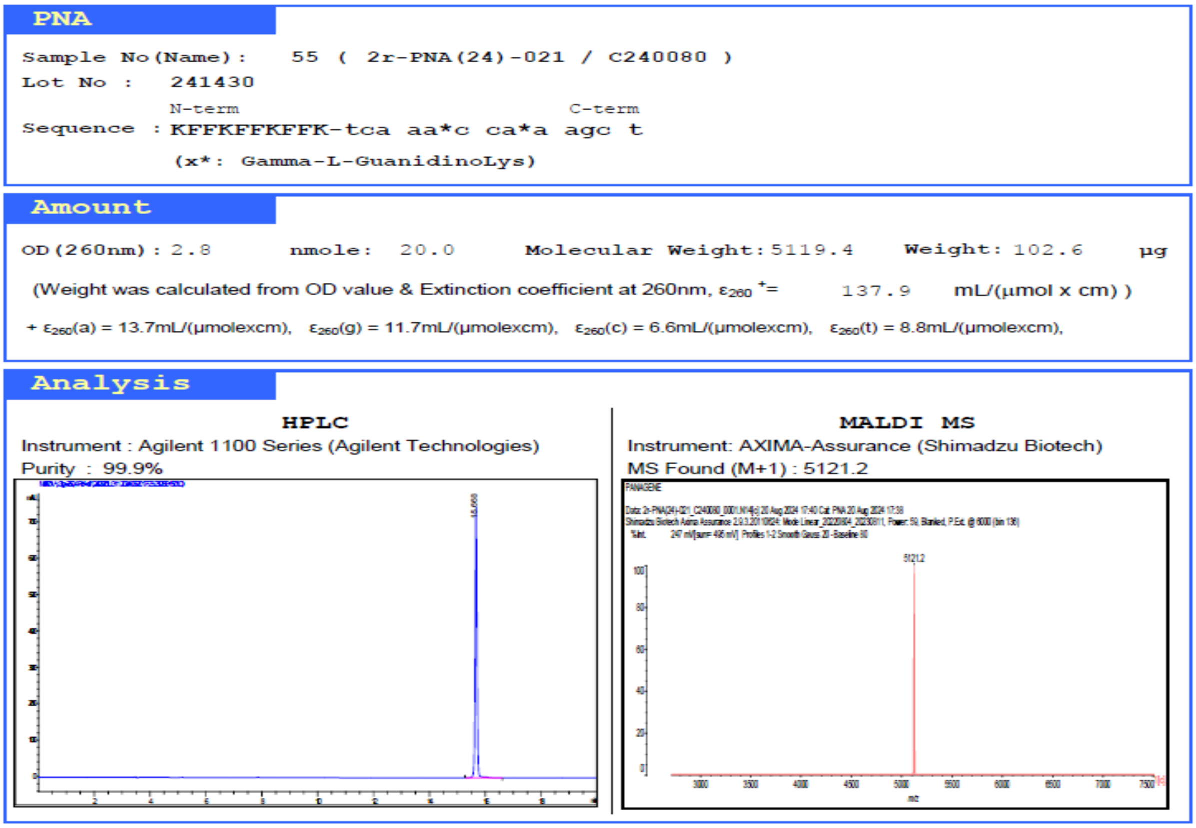

PNA

Sample No(Name) : 55 (2r-PNA(24)-021 / C240080)

Lot No : 241430

Sequence : N-term KFFKFFKFFK-tca aa*c ca*a agc t C-term

(x*: Gamma-L-GuanidinoLys)

Amount

OD(260nm) : 2.8 nmole: 20.0 Molecular Weight: 5119.4 Weight: 102.6 µg

(Weight was calculated from OD value & Extinction coefficient at 260nm, ε_{260} + = 137.9 mL/(µmol x cm))

+ ε_{260}(a) = 13.7mL/(µmolexcm), ε_{260}(g) = 11.7mL/(µmolexcm), ε_{260}(c) = 6.6mL/(µmolexcm), ε_{260}(t) = 8.8mL/(µmolexcm),

Analysis

HPLC

Instrument : Agilent 1100 Series (Agilent Technologies)

Purity : 99.9%

MALDI MS

Instrument: AXIMA-Assurance (Shimadzu Biotech)

MS Found (M+1) : 5121.2

Figure S1. Certificate of Analysis (CoA) results for the α- and γ-modified CPP–PNA conjugants were also obtained from the vendor and are available upon request.

Fmoc-γ-L-GuanidinoLys(diBoc)-PNA-OH	Fmoc-α-D-Arg(Tos)-PNA-OH
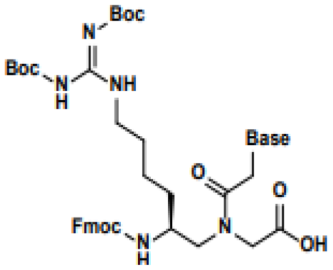	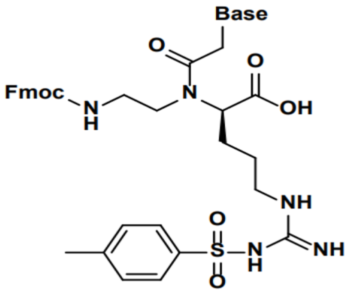

Figure S2. Structural diagram of PNA with α- and γ-modified backbones.

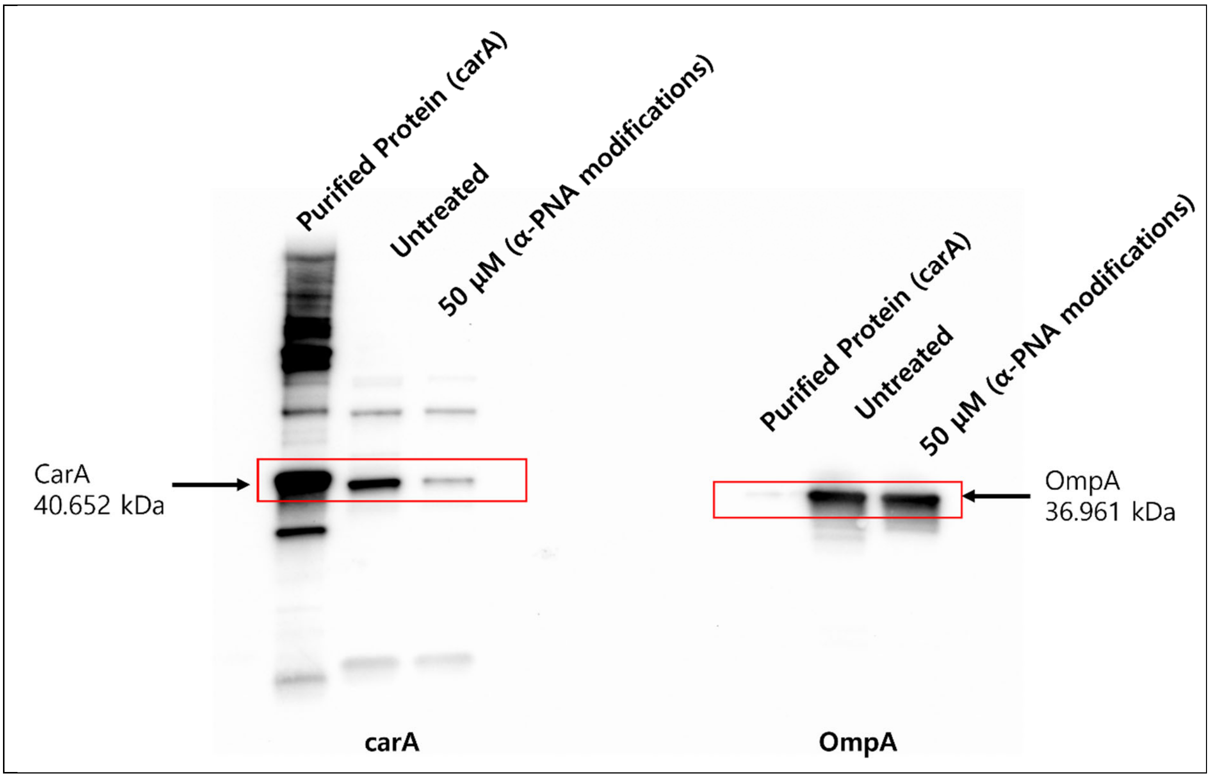

Figure S3. Representative Western blot image of carA protein levels in *A. baumannii* treated with α-PNA modifications targeting the *carA* gene.
